# Endovascular Repair of Type IA Endoleak With Zone 0 TBE and Carotid-Carotid-Subclavian Bypass

**DOI:** 10.1016/j.jaccas.2026.107724

**Published:** 2026-03-27

**Authors:** Anmol K. Multani, Patrick R. Vargo, Francisco B. Alexandrino, Francis J. Caputo, Heba S. Wassif

**Affiliations:** a Department of Cardiology, Heart, Vascular and Thoracic Institute, Cleveland Clinic, Cleveland, Ohio, USA; b Aortic Center, Heart, Vascular and Thoracic Institute, Cleveland Clinic, Cleveland, Ohio, USA; c Department of Thoracic and Cardiovascular Surgery, Cleveland Clinic, Cleveland, Ohio, USA; d Department of Vascular Surgery, Cleveland Clinic, Cleveland, Ohio, USA

**Keywords:** aorta, imaging, postoperative, stents, vascular disease, x-ray fluoroscopy

## Abstract

**Background:**

Type IA endoleaks are a serious complication of thoracic endovascular aortic repair. Consensus is limited on a superior management strategy.

**Case Summary:**

A 76-year-old man with thoracic endovascular aortic repair presented with dyspnea. Imaging revealed a thoracic aortic aneurysm with type IA endoleak compressing adjacent structures. He declined open arch repair and underwent right carotid-left carotid-left subclavian bypass and aortic arch zone 0 thoracic branch endoprosthesis (TBE) placement. The endoleak was eliminated, symptoms resolved, and imaging confirmed graft patency.

**Discussion:**

Type IA endoleaks pose high rupture risk, and zone 0 repair is challenging because cerebral and upper extremity perfusion must be preserved. Evidence for TBE use in zone 0 is sparse. Our case showed successful endovascular carotid-carotid-subclavian bypass with zone 0 TBE placement.

**Take-Home Message:**

Endovascular repair with zone 0 TBE and carotid-carotid-subclavian bypass is a feasible option for type IA endoleak repair.

## Visual Summary

**Figure:**
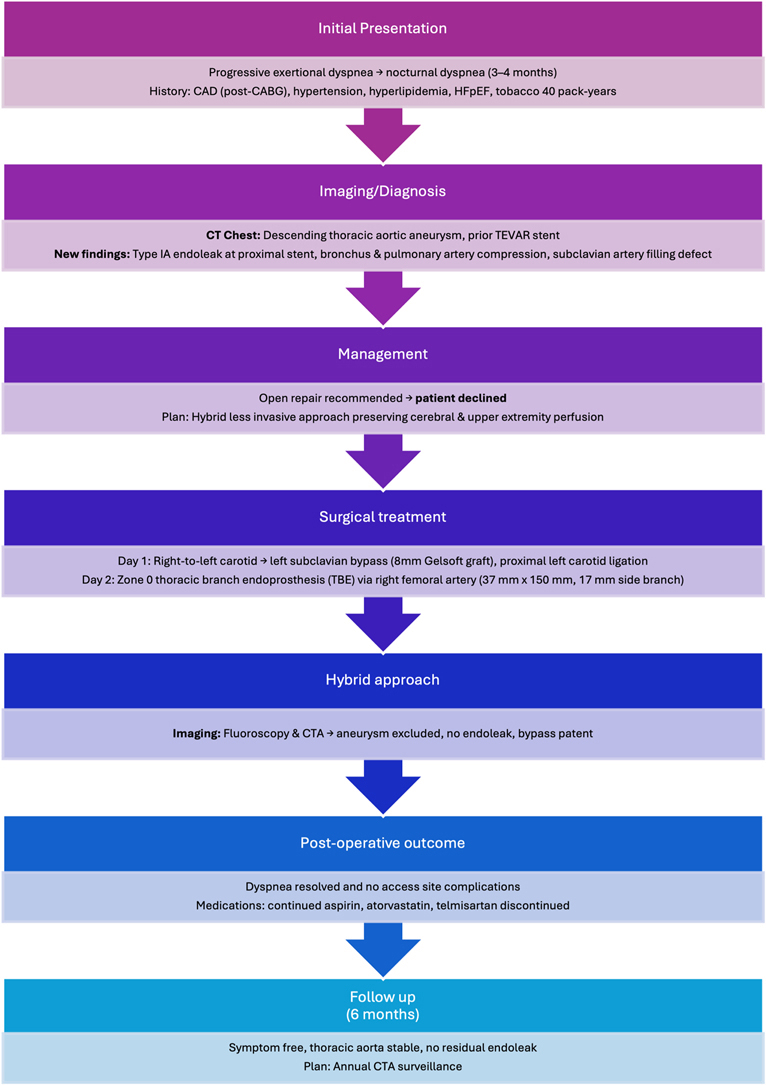

## History of Presentation

A 76-year-old man presented with progressive exertional dyspnea. The respiratory discomfort was initially attributed to COVID-19 infection. He underwent computed tomography of the chest, revealing a large aneurysm in the aortic arch and descending thoracic aorta. The etiology of the aortic aneurysms is likely related to tobacco use (40-pack-year history) and atherosclerosis. No evidence of inflammation was found on imaging or biomarkers. He underwent thoracic endovascular aortic repair (TEVAR), with the stent extending from the aortic arch to the distal descending aorta. On postoperative imaging, an endoleak was found around the beginning of the aneurysm. Although surgical repair was suggested by the vascular surgeon, the patient deferred interventions at that time. The shortness of breath continued to worsen, and he further developed dyspnea nocturnally, requiring 2 pillows for 3 to 4 months. He denied chest pain, cough, lower extremity edema, or syncope. Because of worsening symptoms of dyspnea, he returned to vascular surgery for reconsideration of endoleak repair.

**Take-Home Messages**

• Type IA endoleaks may develop over time after thoracic endovascular aortic repair due to progressive aortic degeneration or failure of the endograft seal and represent a significant clinical challenge requiring prompt repair because of the risk of sac pressurization and rupture.
• The endovascular surgical approach using a thoracic branched endoprosthesis in zone 0 with carotid-carotid-subclavian bypass is a feasible option to manage type IA endoleaks, but they are not as well described.
• Our case adds to the emerging evidence and supports endovascular repair as a viable approach for the elimination of type IA endoleaks.

## Past Medical History

His past medical history was significant for coronary artery disease status post coronary artery bypass grafting. The grafts included the left internal mammary artery to the left anterior descending artery and the diagonal, in situ right internal thoracic artery to the left circumflex artery, and the saphenous vein graft to the posterior descending artery. Other chronic comorbidities were hypertension, hyperlipidemia, and heart failure with preserved ejection fraction.

**Visual Summary:**
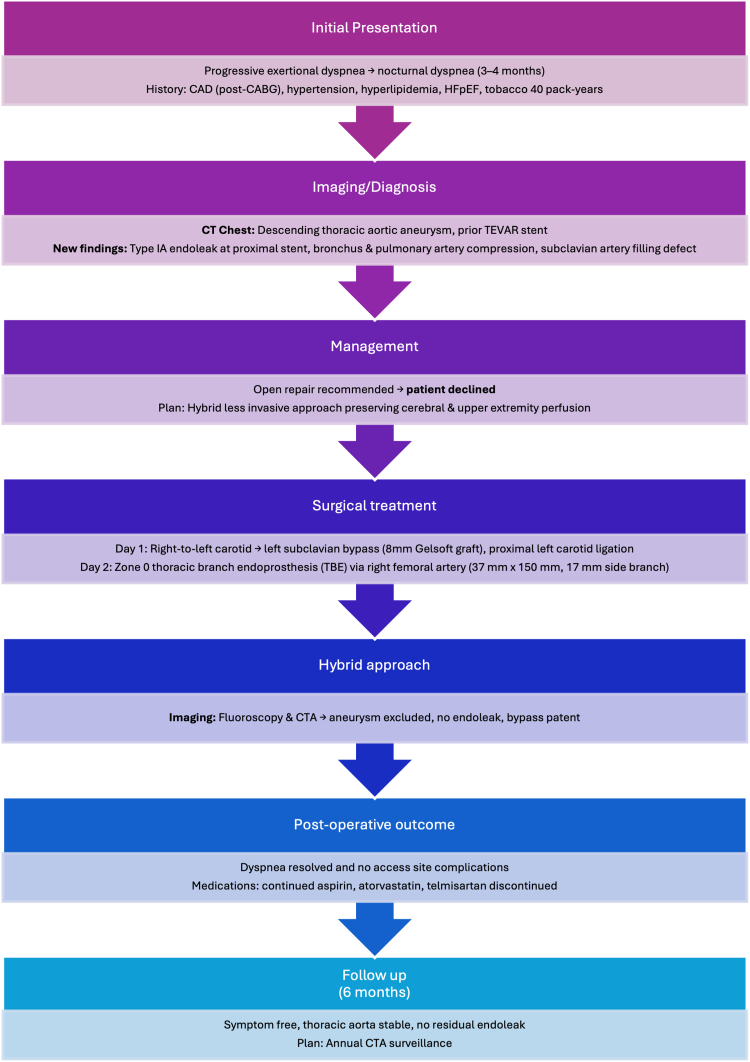
Timeline of Events Visual Summary CABG = coronary artery bypass graft; CAD = coronary artery disease; CTA = computed tomography angiography; HFpEF = Heart failure preserved ejection fraction; TEVAR = thoracic endovascular aortic repair.

## Investigations

**Figure 1:**
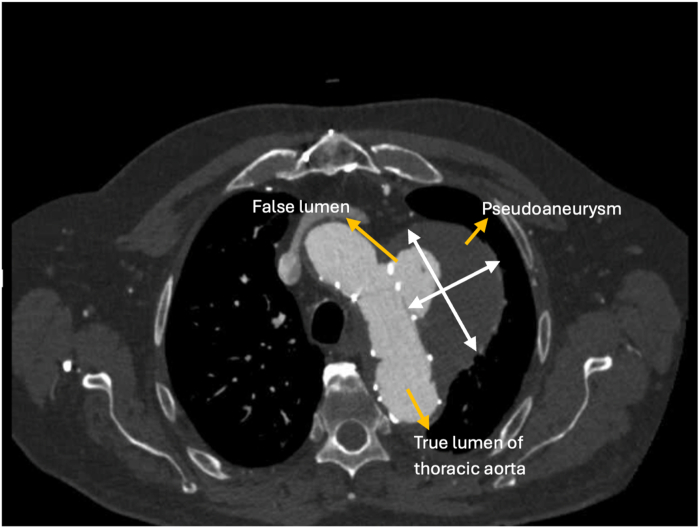
Computed Tomography With Contrast of the Chest Showing an Aortic Aneurysm in the Descending Aorta With a Thoracic Endovascular Aortic Repair Stent

**Figure 2:**
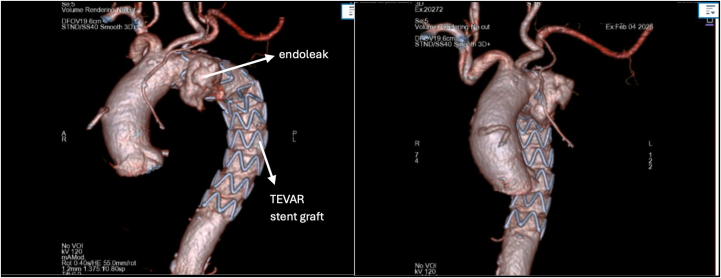
Computed Tomography Angiography Showing an Aortic Aneurysm and TEVAR Stent TEVAR = thoracic endovascular aortic repair.

Computed tomography (CT) with contrast of the chest revealed a large descending thoracic aortic aneurysm with a previously placed TEVAR stent (Figure 1). The endograft extended from just distal to the left common carotid artery, covering the left subclavian artery, and continued into the distal descending thoracic aorta (Figure 2). Although the stent was appropriately positioned through the aneurysmal segment, it failed to completely exclude the aneurysm, leading to the creation of a type IA endoleak at the proximal sealing zone (Figure 2). Contrast enhancement was noted, measuring 4.5 × 3.5 × 2.4 cm. In addition, the aneurysm caused compression of the adjacent bronchus and pulmonary artery, and the stent occluded the left subclavian artery at its origin with distal reconstitution from reversal of vertebral flow.

## Management

**Figure 3:**
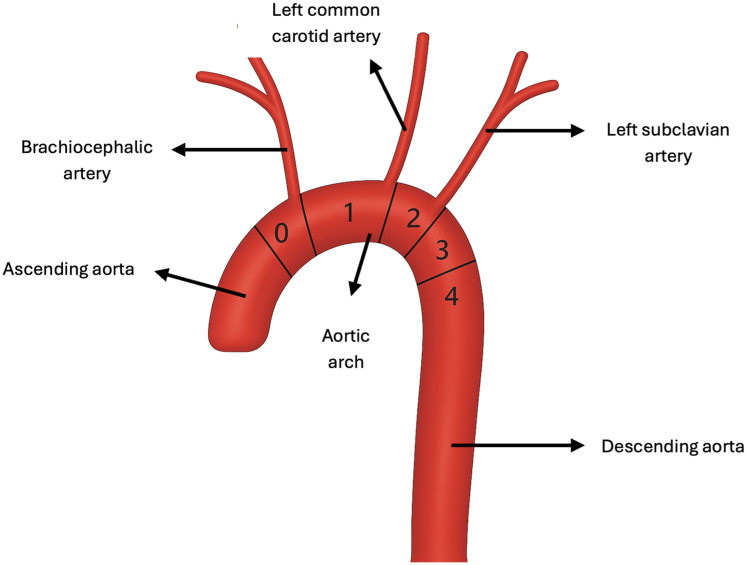
Different Zones of the Aortic Arch

Traditional open surgical arch repair was considered; however, given significant frailty and history of coronary artery bypass surgery, this was estimated to be high risk. Instead, certical debranching followed by zone 0 (Figure 3) stenting of the ascending aorta and arch was performed using the GORE thoracic branch endoprosthesis (TBE) stent.

The patient underwent right common carotid to left common carotid to left subclavian artery bypass using an 8-mm Gelsoft (Terumo Aortic) graft, with ligation of the proximal left common carotid artery. The following day, TBE placement was performed, involving a Gore Tag (Gore) device (37 mm × 150 mm with a 17-mm side branch), via the right common femoral artery.

**Figure 4:**
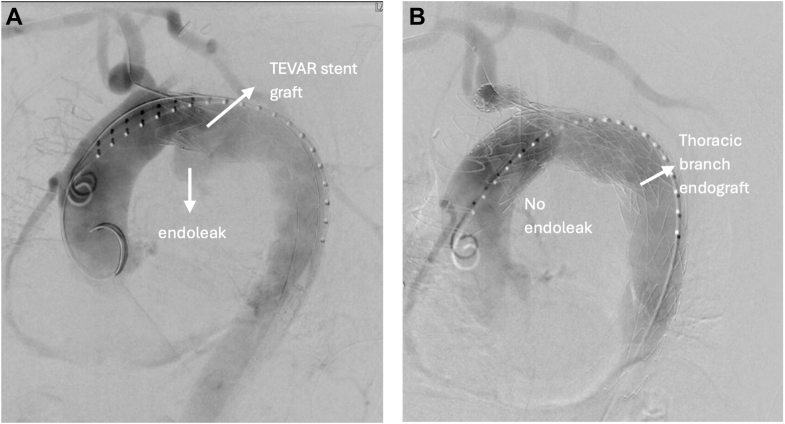
Intraoperative Fluoroscopy Fluoroscopy showing endoleak intraoperatively (A), which was later eliminated with thoracic branched endoprosthesis deployment (B). TEVAR = thoracic endovascular aortic repair.

**Figure 5:**
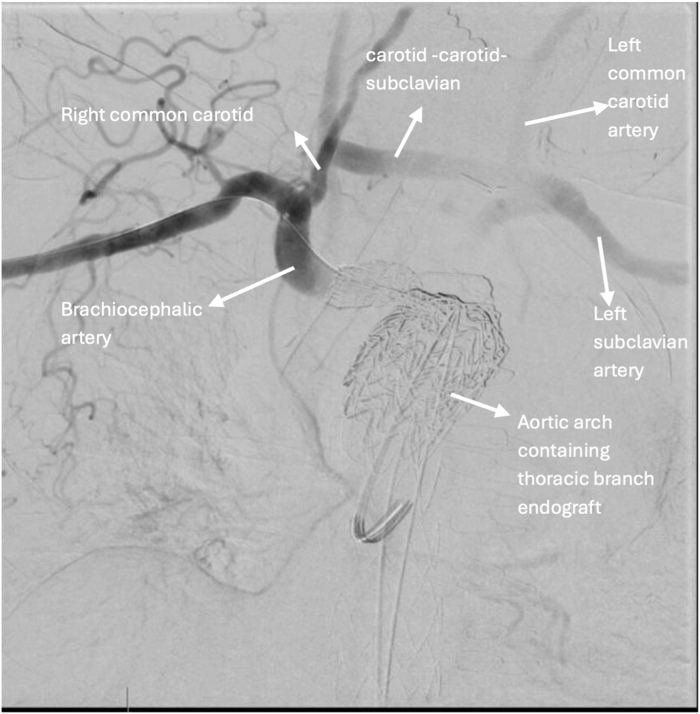
Fluoroscopy Showing Blood Flow Post Carotid-Carotid Bypass and Proximal Extension of Thoracic Endovascular Aortic Repair With a Thoracic Branched Endoprosthesis

Fluoroscopy imaging confirmed successful exclusion of the thoracic aneurysm with no evidence of endoleak post-repair (Figure 3). Adequate blood flow in the carotid and subclavian arteries was also noted on fluoroscopy images post-repair (Figure 4). Duplex ultrasound demonstrated widely patent bilateral carotid, subclavian, and vertebral arteries, with a functioning carotid-carotid-subclavian bypass. Postprocedural CT angiography of the aorta was also performed (Figure 5). No pseudoaneurysm was detected at the right brachial artery access site after hemostasis.

## Outcome and Follow-Up

After the successful procedure, the patient recovered well and was discharged without any complications. His dyspnea resolved completely, and he had no other complaints. The patient continued aspirin 81 mg and atorvastatin 80 mg daily, which were prescribed before the procedure for coronary artery disease. No anticoagulation was recommended. The patient was noted to have elevated blood pressure, and he was prescribed telmisartan before the procedure. However, post-surgery, telmisartan was discontinued as blood pressure during follow-up office visits was well controlled, and he had experienced a few hypotensive episodes. On 6 months of his outpatient follow-up, the patient denied any symptoms, including chest pain or dyspnea. His thoracic aortic measurements were stable, and no endoleak was found on CT angiography of the chest. He will undergo annual CT angiography for surveillance.

## Discussion

TEVAR is being increasingly used to treat thoracic aortic pathologies such as thoracic aortic aneurysms, traumatic aortic injuries, and certain types of aortic dissections.^1^ Even with improving surgical techniques and devices, endoleaks remain a significant complication of these procedures. Type IA endoleaks are concerning because of the high arterial pressure, leading to aneurysm sac pressurization, which increases the risk of continued expansion and rupture.^2^^,^^3^ Guidelines suggest that endoleaks are not only the most common complication with an incidence of 23% to 32%,^4^ but also the most dangerous complication of TEVAR with a mortality rate of 90% in cases of acute rupture.^3^ Prompt management is indicated because of the risk involved.^2^

Treatment options have expanded to endovascular techniques such as fenestrated or branched stent grafts to proximally extend the stent graft,^5^ endostapling or endoanchors,^6^ chimney grafts, and embolization procedures.^7^ In addition, balloon molding and placement of bare stents to strengthen the graft at the proximal zone are other treatment options.^8^ There is consensus on the prompt treatment of type IA endoleaks in the literature; however, there is limited evidence to suggest that one treatment strategy is betterthan others for type IA endoleak repair.^5^

Our case demonstrates the utility of thoracic single-branched endoprostheses to eliminate type IA endoleaks by extending TEVAR into zone 0 of the aortic arch. Zone 0 is a complex anatomical site as supra-aortic vessels are involved and cerebral and upper extremity perfusion needs to be preserved.^9^ Our case highlights that this can be achieved with an endovascular approach with TBE using carotid-subclavian bypass. The extra-anatomic bypass allowed circulation in the left subclavian artery and contralateral cerebral circulation via the left common carotid artery.

The use of TBEs, especially in proximal aortic arch pathology up to zone 0, is not well studied.^10^ An analysis by Erben et al^9^ showed that TBE use in zone 0 of the aortic arch is safe and effective for type A dissection. In addition, there was a postoperative observational study that showed favorable 3-year outcomes of GORE TAG endoprosthesis with carotid-subclavian bypass for aortic arch aneurysms.^10^ There are limited reported cases of using this technique of endovascular zone 0 TBE and carotid-carotid-subclavian bypass for a type IA endoleak; this case adds to the growing body of evidence supporting the application of this technique.

A less invasive endovascular approach was used for this patient based on the patient’s preferences. Although durability with stents placed in endovascular repair can be a limitation, the patient has not had any complications postoperatively. Our patient’s bypass remained patent on postoperative imaging, and the aneurysm was successfully excluded with no residual endoleak or access site complications.

## Conclusions

Type IA endoleaks are a serious complication of TEVAR and can lead to rupture of the aneurysm sac with ongoing pressurization of the sac. Management requires an individualized approach, with open and endovascular strategies serving as 2 potential approaches. A hybrid approach of deployment of a thoracic branched endoprosthesis in zone 0 of the aortic arch with carotid-carotid-subclavian bypass was used in our patient and had a successful outcome of endoleak elimination with a lower risk of mortality and morbidity. Our successful outcome, supported by existing but limited literature, highlights the potential of this endovascular strategy as a viable option in complex aortic arch pathology and the need for further studies to establish its long-term efficacy.

## Funding Support and Author Disclosures

All data were collected and analyzed independently at the Cleveland Clinic Main Campus. The authors have reported that they have no relationships relevant to the contents of this paper to disclose.
